# The use of ambient humidity conditions to improve influenza forecast

**DOI:** 10.1371/journal.pcbi.1005844

**Published:** 2017-11-16

**Authors:** Jeffrey Shaman, Sasikiran Kandula, Wan Yang, Alicia Karspeck

**Affiliations:** 1 Department of Environmental Health Sciences, Mailman School of Public Health, Columbia University, New York, NY, United States of America; 2 National Center for Atmospheric Research, Boulder, CO, United States of America; University of Chicago, UNITED STATES

## Abstract

Laboratory and epidemiological evidence indicate that ambient humidity modulates the survival and transmission of influenza. Here we explore whether the inclusion of humidity forcing in mathematical models describing influenza transmission improves the accuracy of forecasts generated with those models. We generate retrospective forecasts for 95 cities over 10 seasons in the United States and assess both forecast accuracy and error. Overall, we find that humidity forcing improves forecast performance (at 1–4 lead weeks, 3.8% more peak week and 4.4% more peak intensity forecasts are accurate than with no forcing) and that forecasts generated using daily climatological humidity forcing generally outperform forecasts that utilize daily observed humidity forcing (4.4% and 2.6% respectively). These findings hold for predictions of outbreak peak intensity, peak timing, and incidence over 2- and 4-week horizons. The results indicate that use of climatological humidity forcing is warranted for current operational influenza forecast.

## Author summary

Laboratory and epidemiological evidence indicate that atmospheric absolute humidity conditions modulate the survival, transmission, incidence and seasonality of influenza. Absolute humidity (AH) conditions are often incorporated as a forcing factor in mathematical models used to describe and forecast influenza incidence. Here we examine whether the inclusion of absolute humidity forcing improves influenza forecast accuracy. We perform retrospective influenza forecasting over 10 seasons for 95 cities using 4 different forms of AH forcing: 1) no AH forcing; 2) optimization and forecast with local climatological AH forcing; 3) optimization and forecast with local observed AH forcing; and 4) optimization with observed AH forcing and forecast with climatological AH forcing. We find that humidity forcing improves forecast performance and that forecasts generated using climatological humidity forcing generally outperform forecasts that utilize observed humidity forcing.

## Introduction

A growing body of evidence indicates that the survival and transmissibility of influenza are affected by ambient humidity conditions. Laboratory experiments have shown that aerosolized influenza survival rates increase in low ambient relative humidity (RH), i.e. less than 40% [1–10]. Additional experiments examining the transmission of human influenza among guinea pigs have also shown that transmission increases at low RH levels [11]. Further evaluation of these experiments has revealed a strong relationship in which low *absolute* humidity (AH) conditions favor the survival and transmission of influenza [12].

Low AH conditions manifest outdoors during winter in temperate regions. Furthermore, because temperature indoors is managed but humidity is generally not, both AH and RH tend to be low indoors during winter. Indeed, even though RH is often maximal outdoors during winter, indoor RH often reaches the very low levels (10–40%) favorable for influenza survival and transmission, and indoor AH closely mirrors outdoor levels [13–16].

People in the developed world, such as the US, spend approximately 90% of their time indoors [17]; as a result, influenza transmission is suspected to occur indoors in the developed world. In addition, because both indoor RH and indoor AH are highly co-variable with outdoor AH, either variable can be estimated using *outdoor* AH. Consequently, regardless of whether one considers RH or AH to be the true modulator of influenza survival and transmissibility (and the mechanisms for this modulation remain undetermined), outdoor AH can be used to estimate the effect of humidity on influenza transmission.

A number of epidemiological studies have found associations between outdoor AH and estimates of influenza incidence or influenza-related mortality. Statistical analysis has shown that the onset of influenza outbreaks is associated with anomalously low AH conditions [18]. Low AH levels in temperate regions have also been associated with increased influenza-associated mortality levels [19], increased influenza transmission intensity [20], and increased influenza incidence [21].

Modeling studies have shown that the seasonality of influenza in temperate regions can be reproduced when influenza transmission potential is modulated by observed AH conditions [18]. Furthermore, AH, along with other dynamical processes, can be used to explain the timing of both seasonal and pandemic influenza outbreaks [22–25]. Indeed, even the development of pandemic influenza outbreaks out of season (i.e. during summer) can be understood in the context of ambient seasonal humidity conditions, contact patterns, and population susceptibility. Specifically, ambient humidity sets an upper bound on the transmission potential (a maximal basic reproductive number), contact patterns also influence transmission (e.g. through preferential mixing among certain sub-populations), and population susceptibility reduces transmissibility as it drops. In effect, AH constrains the extent to which an influenza virus strain is capable of sustained transmission during summer and sustained transmission is possible only if population susceptibility to that strain remains sufficiently high. For circulating seasonal influenza strains, higher AH and lower susceptibility conspire to limit sustained transmission of circulating influenza strains during summer; however, the introduction of a new pandemic strain, for which population susceptibility is much higher, can enable sustained transmission during summer [22, 25].

Recently, we developed a number of model-inference systems for the ensemble forecast of seasonal influenza [26–29]. These systems use a compartmental model, such as a susceptible-infected-recovered-susceptible (SIRS) model, that is first optimized prior to forecast using observational estimates of US state and municipal influenza incidence. When first developed, we used an AH-forced SIRS model for these predictions, as this model had been used to describe the seasonality of influenza at state geographic scales in the US [18]. However, when applied to influenza forecast over larger areas, e.g. US CDC Health and Human Services multi-state regions, or subtropical regions, we have heretofore adopted a SIRS form without AH forcing [27, 30]. It remains an open question whether the prediction of influenza in temperate regions is improved by the inclusion of AH forcing. In this paper, we perform retrospective forecasting over 10 seasons for 95 cities using 4 different forms of AH forcing: 1) no AH forcing; 2) optimization and forecast with local climatological AH forcing (i.e. historical average AH conditions on a given day); 3) optimization and forecast with local observed AH forcing (i.e. AH as observed on a given day); and 4) optimization with observed AH forcing and forecast with climatological AH forcing. This effort applies these 4 AH forcing approaches to an established model-inference prediction system that has been used for 5 years to forecast influenza operationally in real-time [27, 31]. We explore whether clear differences in forecast accuracy emerge among these 4 approaches and quantify whether inclusion of AH forcing improves forecast accuracy. We hypothesize, given evidence suggesting ambient AH modifies the survival and transmissibility of influenza, that AH forcing will improve dynamic model influenza forecast.

## Methods

### Modeling approaches

Forecast of influenza is here generated using compartmental models describing the propagation of influenza through a population, observational estimates of influenza incidence, and data assimilation methods for model optimization [26–29]. Four different compartmental models were used to generate the forecasts. All four forms are perfectly-mixed, absolute humidity-driven compartmental constructs with the following designations: 1) susceptible-infectious-recovered (SIR); 2) SIRS; 3) susceptible-exposed-infectious-recovered (SEIR); and 4) susceptible-exposed-infectious-recovered-susceptible (SEIRS). The differences among the model forms align with whether waning immunity, which allows recovered individuals to return to the susceptible class, or an explicit period of latent infection (the exposed period) is represented.

As the SEIRS model is the most detailed, we present it here. All other forms are derived by reduction of these equations, which are as follows:

$$\frac{dS}{dt}=\frac{N-S-E-I}{L}-\frac{\beta (t)IS}{N}-\alpha$$ (1)

$$\frac{dE}{dt}=\frac{\beta (t)IS}{N}-\frac{E}{Z}+\alpha$$ (2)

$$\frac{dI}{dt}=\frac{E}{Z}-\frac{I}{D}$$ (3)

where *S* is the number of susceptible people in the population, *t* is time in years, *N* is the population size, *E* is the number of exposed people, *I* is the number of infectious people, *N*-*S*-*E*-*I* is the number of recovered individuals, *β*(*t*) is the contact rate at time *t*, *L* is the average duration of immunity, *Z* is the mean latent period, *D* is the mean infectious period, and α is the rate of travel-related import of influenza virus into the model domain.

The contact rate, *β*(*t*), is given by *β*(*t*) = *R*_0_(*t*)/*D*, where *R*_0_(*t*), the basic reproductive number, is the number of secondary infections the average infectious person would produce in a fully susceptible population at time *t*. Specific humidity, a measure of absolute humidity (AH), modulates transmission rates within this model by altering *R*_0_(*t*) through an exponential relationship derived from laboratory experiments [12]:

$$R_{0}(t)=R_{0\min}+\left(R_{0\max}-R_{0\min}\right)e^{-aq(t)}$$ (4)

where *R*_0min_ is the minimum daily basic reproductive number, *R*_0max_ is the maximum daily basic reproductive number, *a* = 180 (unitless), and *q*(*t*) is the time-varying specific humidity (in kg/kg). The value of *a* is estimated from the laboratory regression of influenza virus survival upon AH [18]. Simulations were performed with fixed travel-related seeding of *I* of 0.1 infections per day (1 infection every 10 days).

For each of the 4 compartmental model forms (SIR, SIRS, SEIR, SEIRS), four different approaches were used to test how the incorporation of AH conditions in the model framework affects the accuracy of influenza forecast. Each approach applied AH conditions in a different fashion. In the first approach, ‘observed AH’, observed local daily AH conditions are applied using Eq 4. This use of AH is not realistic for real-time forecast, as future AH conditions are not known; however, as applied here, it can be used to forecast influenza outbreak characteristics retrospectively and to determine if such information would, in theory, improve forecast accuracy.

For the second approach, ‘climatological AH’, we use local daily climatological AH conditions in Eq 4. Here, the climatology is based on 24 years (1979–2002) and represents the historical average conditions on a given day for the location at which the model is applied. Due to averaging over many years, climatological AH is much smoother than observed AH conditions and has been used operationally to generate real-time influenza forecasts [27, 31].

The third approach, ‘combination AH’, is a hybrid of the first two approaches. Observed AH is used during model optimization prior to forecast, but the forecast of future outcomes is generated using climatological AH. This strategy can be used for real-time forecasting and was implemented previously [26].

The final approach, ‘no AH’, replaces Eq 4 with *R*_0_(*t*) = *R*_0_. In this fashion *R*_0_ is treated as an adjustable parameter to be optimized during the data assimilation process (see below). As with the other parameters of the model, it remains fixed during forecast when the optimized model is integrated into the future to generate an ensemble of predictions.

### Data

Specific humidity (*q*; used in Eq 4) data were compiled from the National Land Data Assimilation System (NLDAS) project-2 dataset. These data are derived through spatial interpolation, temporal disaggregation and vertical adjustment from station measurements and National Center for Environmental Prediction North American Regional Reanalysis [32]. The gridded NLDAS meteorological data are available in hourly time steps on a 0.125° regular grid from 1979 through the present [33]. Specific humidity data from the grid cell containing the centroid of each of the 95 cities included in this study were assembled for 1979–2015. These hourly data were then averaged to daily resolution. A 1979–2002 (24 year) daily climatology was then constructed for each city.

As described in Shaman et al. [27], weekly estimates of influenza incidence were generated by multiplying 2003–2015 historical Google Flu Trend (GFT) estimates of municipal influenza-like illness (ILI) [34], as these data were released in real time (S1 Data), by coincident census division (regional) weekly laboratory-confirmed influenza positive proportions as compiled through the National Respiratory and Enteric Virus Surveillance System (NREVSS) and U.S.-based World Health Organization (WHO) Collaborating Laboratories [35]. This combined metric, termed ILI+, provides a more specific measure of influenza incidence than ILI alone, which non-specifically captures signal from other circulating respiratory viruses, such as adenovirus and rhinovirus [27, 36]. While the spatially coarser regional NRVESS/WHO data does not fully discriminate variability in influenza positivity at the municipal scale, multiplication of municipal ILI with these regional proportions does remove some of the signal associated with other respiratory viruses and provides a more precise estimate of influenza activity than ILI alone.

### Data assimilation

Three ensemble filter methods—the ensemble Kalman filter [37], the ensemble adjustment Kalman filter [38] and the rank histogram filter [39]—and a particle filter (PF) with resampling and regularization [40] were used in conjunction with ILI+ to optimize and initialize the compartmental models prior to forecast. Both the model state variables (*S*, *E*, *I*) and parameters (*L*, *Z*, *D*, *R*_0max_, *R*_0min_ and *R*_0_) were subject to optimization. Ensemble filter simulations were run with 300 ensemble members and PF simulations were run with 10,000 particles.

Each ensemble filter algorithm is applied sequentially through time to update ensemble model simulations of observed state variables (i.e. influenza incidence) to better align with observations (i.e. ILI+). These updates are calculated, per the specifics of each filter algorithm (described below), by halting the ensemble integration when a new observation comes available. The posterior is then integrated through time using the model equations to the next observation and the process is repeated. Through this iterative updating process the ensemble of simulations provides an increasingly accurate estimate of the observed state variable (i.e. influenza incidence), and estimates of the unobserved variables and parameters (e.g. susceptibility and mean infectious period) are obtained through additional adjustments that take advantage of their co-variability with the observed state variable.

In general, Kalman filters assume normality of the observational error, the prior distribution, and the posterior distribution. Differences among the ensemble filter algorithms manifest from the means by which the update is specified. The ensemble Kalman filter (EnKF) is a stochastic, perturbed observation Kalman filter in which the update of each ensemble member is computed using the current observation plus Gaussian random noise [41]. That is, the posterior for each ensemble member is simply the weighted sum of the prior for that ensemble member and the observation plus random noise with variance equal to the observational error variance. The weights themselves are calculated as ratios of the ensemble prior variance and the observational error variance.

The ensemble adjustment Kalman filter (EAKF) employs a deterministic algorithm to compute the ensemble posterior mean and variance [38]. At each update, the EAKF algorithm aligns the first two ensemble posterior moments with those predicted by Bayes’ theorem.

Unlike the EnKF and EAKF, the rank histogram filter (RHF) does not impose a Gaussian structure on the prior, observations or posterior [39]; rather, this filter employs an algorithm that creates an approximate probability distribution by ordering (i.e. ranking) the ensemble prior. In this fashion, the RHF admits non-Gaussian distributions, thus relaxing the normality assumption inherent to most Kalman filters.

For the ensemble filters, multiplicative inflation [38] was applied following the assimilation of each weekly observation of ILI+. The inflation was used to counter the ensemble filter tendency toward ‘filter divergence', which occurs when the prior ensemble spread becomes spuriously low. In the absence of inflation, the system may give too little weight to the observations and thus diverge from the true trajectory.

Unlike the above ensemble filters, PFs are an alternate class of assimilation method that do not require assumptions about linearity or normality. The PF approach used here adopts sequential importance sampling with resampling and regularization [40, 42]. Resampling generates a new suite of particles with equal weight during the model integration whenever the effective sample size is low. Regularization jiggles the state and parameter values of each resampled particle to eliminate redundancies and further sample parameter space around each previously highly weighted particle. As a consequence of resampling and regularization, a much richer range of parameter and state space is spanned than with a basic PF, which relies only on the initial parameter choices.

For all 16 model-filter combinations (i.e. 4 models × 4 filters) a scaling factor was employed to convert ILI+ to influenza incidence, the quantity represented in the compartmental models, per Shaman et al. [27]. Both ILI and ILI+ are biased in that they only capture persons seeking medical attention and are measured per 100,000 patient visits. The scaling factor partially compensates for this bias by accounting for the probability that a person with influenza seeks medical attention and the probability that a person seeks medical attention for any reason [27]. Even after this scaling, bias in the data likely remains; however, as long as this bias remains stationary, it should not corrupt forecasting. That is, if a given model is well optimized using biased observations, it should make biased predictions, as the accuracy of those out-of-sample predictions is being assessed using the same biased dataset.

Additional details on the application of the ensemble filters and PF to infectious disease models are provided in Shaman and Karspeck [26] and Yang et al. [28]. S1–S3 Figs present example forecasts from the more than 1.2 million predictions generated.

### Forecast metrics

For each model-filter combination, we generated weekly retrospective forecasts of influenza outbreak characteristics during weeks 6–25 of the influenza season (beginning early October) over 10 seasons (2003–2004 through 2014–2015, excluding the pandemic seasons 2008–2009 and 2009–2010). For the ensemble filters, the ensemble mean trajectory was used for forecast accuracy assessment; for the PF, the particle weighted average trajectory was used. A simple average of these trajectory forecasts as generated by all 16 model-filter combinations was used for accuracy analysis unless otherwise specified. We limit our analysis to forecasts made 0–8 weeks before the predicted peak week.

Let *O*(*t*) be the ILI+ observed at time *t* and *F_w_*(*t*) the ILI+ forecast made for time *t* using ILI+ available through week *w*, i.e. *w* < *t*. The predicted peak intensity at *w* is defined as the maximum of the average forecast trajectory, and the peak week is the week when that maximum occurs. A predicted peak week is defined as *accurate* if it is within ±1 week of the observed peak week, and the predicted peak intensity is deemed accurate if it is within ± 25% of the observed peak intensity. In addition, absolute error was calculated for each prediction of peak week and peak intensity and used to rank the weekly performances of the 4 AH forecast approaches.

Root mean squared error (RMSE) was calculated over the entire trajectory at time horizons of 2 and 4 weeks:

$$RMSE_{w}^{h}=\frac{\sqrt{\sum_{t=w+1}^{w+h}{\left(F_{w}(t)-O(t)\right)}^{2}}}{h}$$ (5)

where *h* ∈ {2, 4} weeks. We also performed a Friedman test followed by a Nemenyi test to assess whether forecast error differed significantly among the 4 AH forcing approaches. The Friedman test is a non-parametric test that ranks the error of each group—here, each AH forcing approach—for each forecast location-week. The Nemenyi test assesses for statistically significant differences between each pair of ranked groupings.

## Results

**Fig 1:**
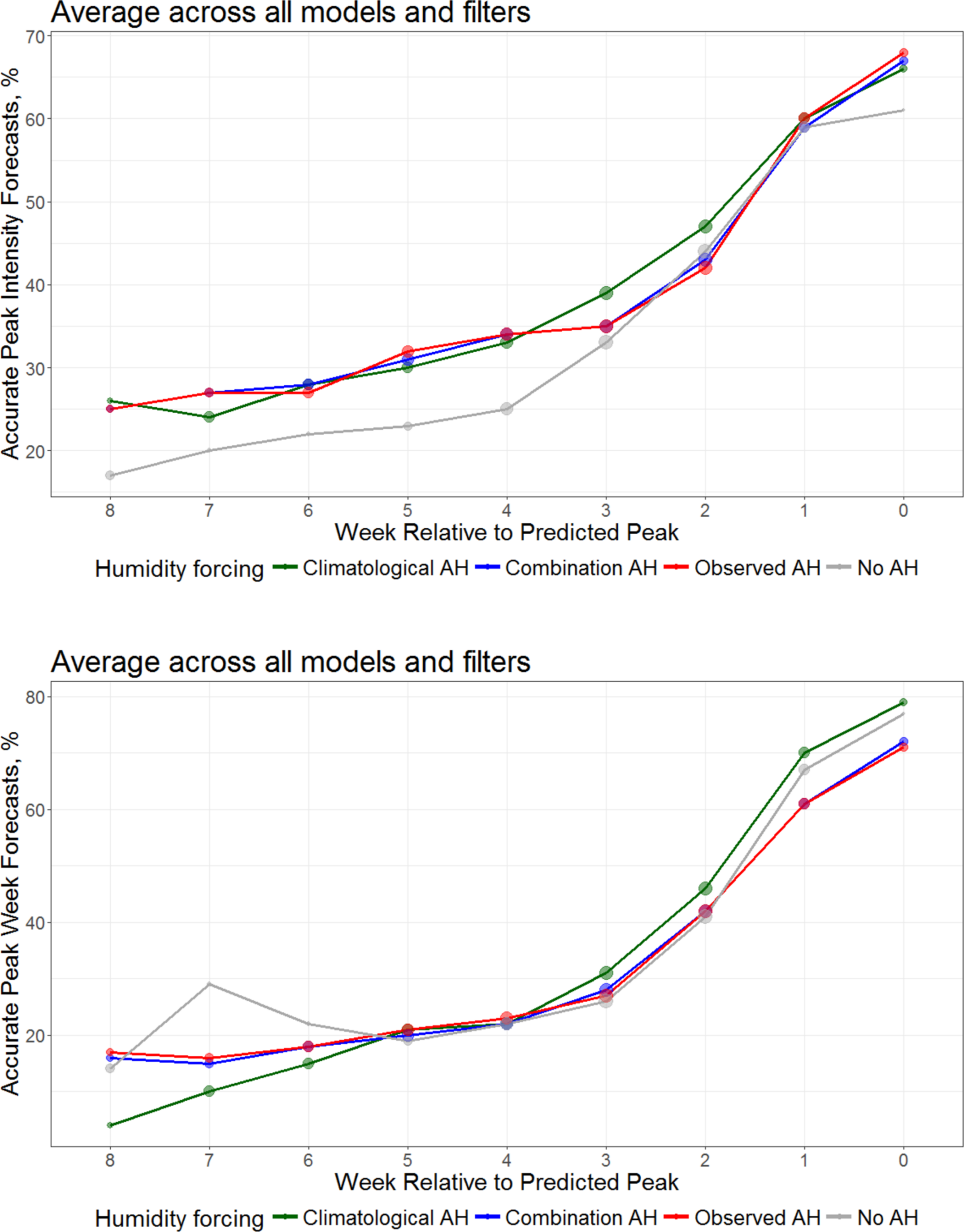
Percentage of forecasts accurate for predictions of peak intensity (top, within ±25% of observed peak intensity) and peak timing (bottom, within ±1 week of the observed peak) plotted as a function of forecast lead relative to the predicted peak. Shown are the forecast accuracies for models with climatological AH forcing (green), observed AH forcing (red), a combination of observed AH during optimization and climatological AH during forecast (blue), and no AH forcing (grey). The number of forecasts (log transformed) at each lead is represented by the size of the dot.

For predictions of peak intensity, forecasts using one of the 3 AH forcing approaches were superior at all lead times (**Fig 1**). Peak week forecasts showed similar results, with the exception of the forecasts with 6- and 7-week leads. Note that the number of no AH forecasts generated with 6- and 7-week leads was small, so this result may be due to sampling error. The climatological AH forecasts were most accurate for leads of 0 through 3 weeks. These findings were insensitive to the choice of error margin used to define accuracy (S4 and S5 Figs).

**Fig 2:**
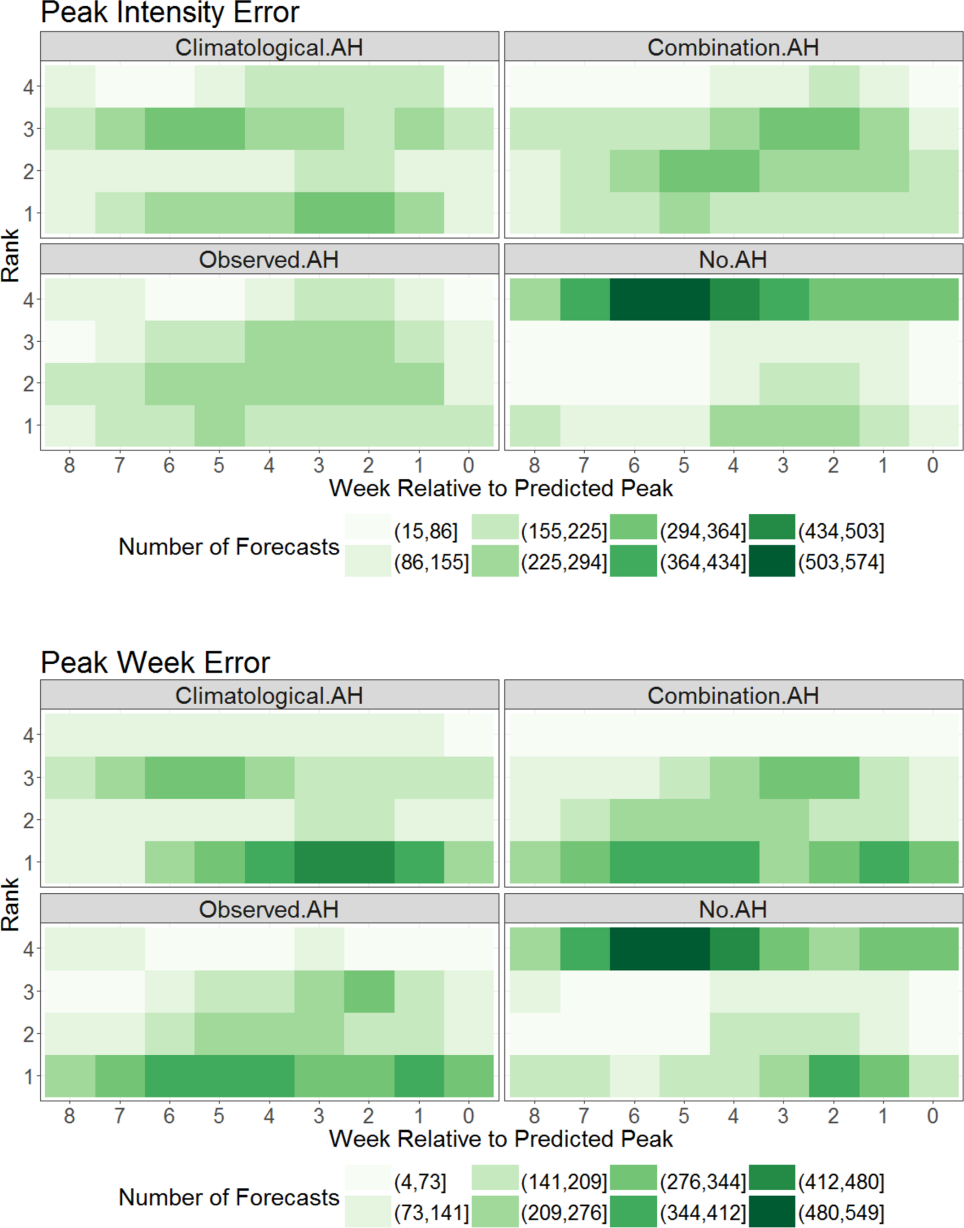
Heat map of forecast error rank for predictions of peak intensity (top) and peak timing (bottom) plotted as a function of forecast lead relative to the predicted peak. Weekly forecasts for a location were ranked (1–4) based on prediction error for a given metric, where 1 was the forecast with the least error. Color indicates the number of forecasts at each lead with a given error ranking relative to the other forms. Darker colors indicate more forecasts at a given lead with a particular ranking.

Forecast error rank for peak intensity reveals the climatological AH forecasts most often had the lowest rank (smallest error) at most leads and the no AH forecasts most often had the largest error at all leads (**Fig 2**). For predictions of peak week, the no AH forecasts again most often had the largest error at all leads; however, differences among the 3 humidity-forced forecasts were less clearly discernible. These findings were confirmed by Friedman rank, which showed that overall the no AH forecasts ranked the worst among the four AH approaches (**Table 1**). Results from the pairwise comparison revealed highly significant differences among most pairings (p < 0.001) with the exception of the climatological AH-combination AH pair (**Table 2**).

**Table 1:** Mean Friedman ranks of forecast error for predictions of peak intensity, peak week and incidence during the first 2 weeks (RMSE2) and 4 weeks (RMSE4) of forecast. For pairwise tests of significance see Table 2. Best performing model forms are in bold. Note, two forms may be best if not statistically different.

Forecast	Peak Intensity	Peak Week	RMSE2	RMSE4
**Climatological AH**	**2.36**	**2.33**	**2.35**	**2.36**
**Combination**	**2.32**	**2.34**	2.42	2.43
**No AH**	2.89	2.88	2.69	2.64
**Observed AH**	2.43	2.45	2.54	2.56

**Table 2:** Pairwise p-values derived from Nemenyi tests of the forecast ranks shown in Table 1. Asterisks designate differences significant at p<0.01 (**) and p<0.001 (***).

**Peak Intensity Forecasts**				**Peak Week Forecasts**			
	**Climatological**	**Combination**	**No AH**		**Climatological**	**Combination**	**No AH**
**Combination**	0.234	-	-	**Combination**	0.899	-	-
**No AH**	<0.001***	<0.001***	-	**No AH**	<0.001***	<0.001***	-
**Observed**	<0.01**	<0.001***	<0.001***	**Observed**	<0.001***	<0.001***	<0.001***
**RMSE during the first 2 weeks of Forecast (RMSE2)**				**RMSE during the first 4 weeks of Forecast (RMSE4)**			
	**Climatological**	**Combination**	**No AH**		**Climatological**	**Combination**	**No AH**
**Combination**	0.003**	-	-	**Combination**	0.003**	-	-
**No AH**	<0.001***	<0.001***	-	**No AH**	<0.001***	<0.001***	-
**Observed**	<0.001***	<0.001***	<0.001***	**Observed**	<0.001***	<0.001***	0.001***

**Fig 3:**
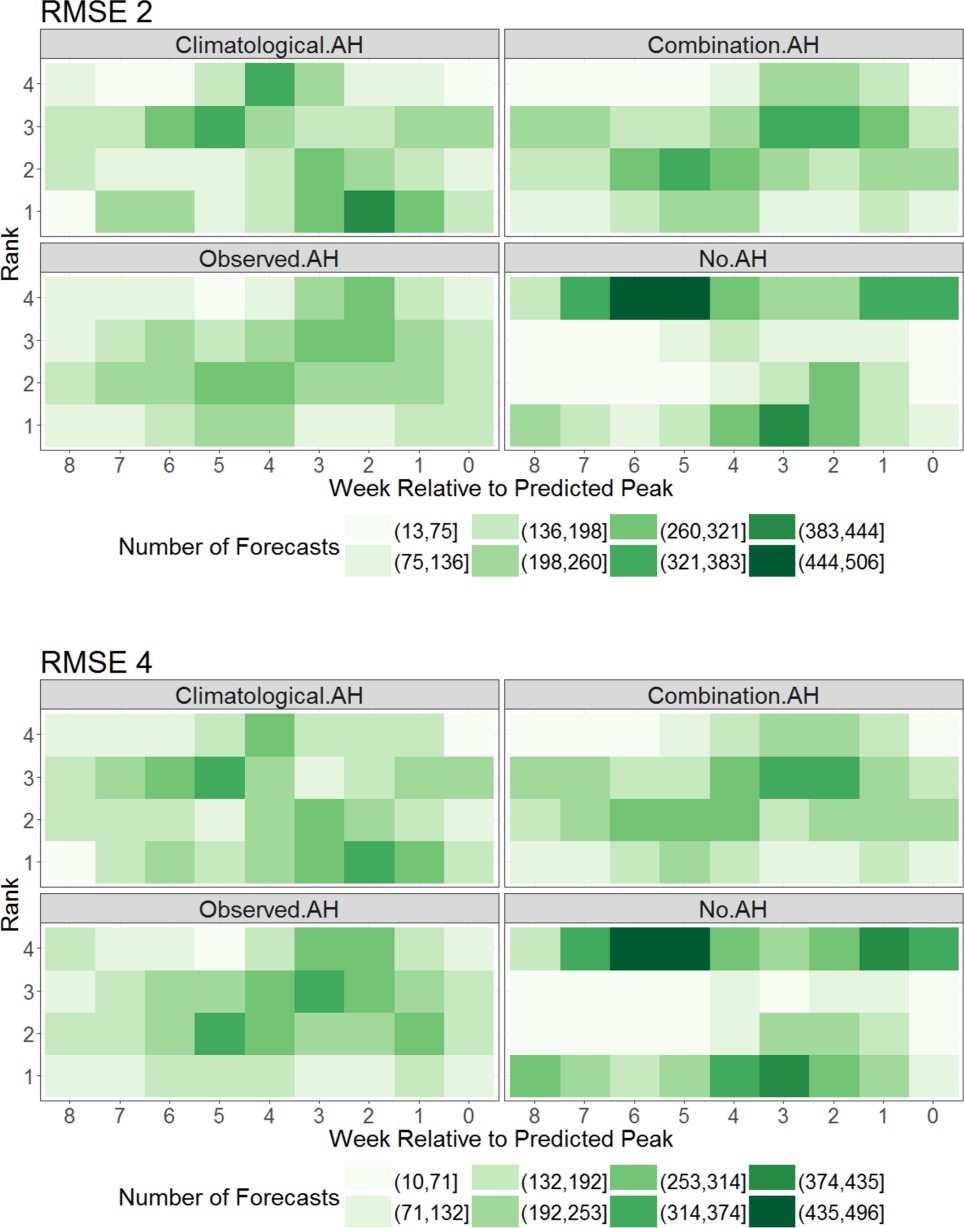
As for Fig 2, but showing RMSE of incidence for the first 2 weeks of forecast (top, RMSE 2) and the first 4 weeks (bottom, RMSE 4).

Similar findings hold for RMSE over 2- and 4-week prediction horizons (**Fig 3**). Here, the no AH forecasts rank either best or worst depending on lead time. Specifically, the no AH forecasts rank worst at longer leads but often rank best at shorter leads (2 to 4 weeks). In contrast, the combination and observed AH forecasts most often cluster at ranks 2 and 3. The climatological AH forecasts appear to have the greatest diversity of ranking; however, overall it has the lowest mean ranking (**Table 1**) and provides a significant benefit over the 3 other approaches (**Table 2**).

**Fig 4:**
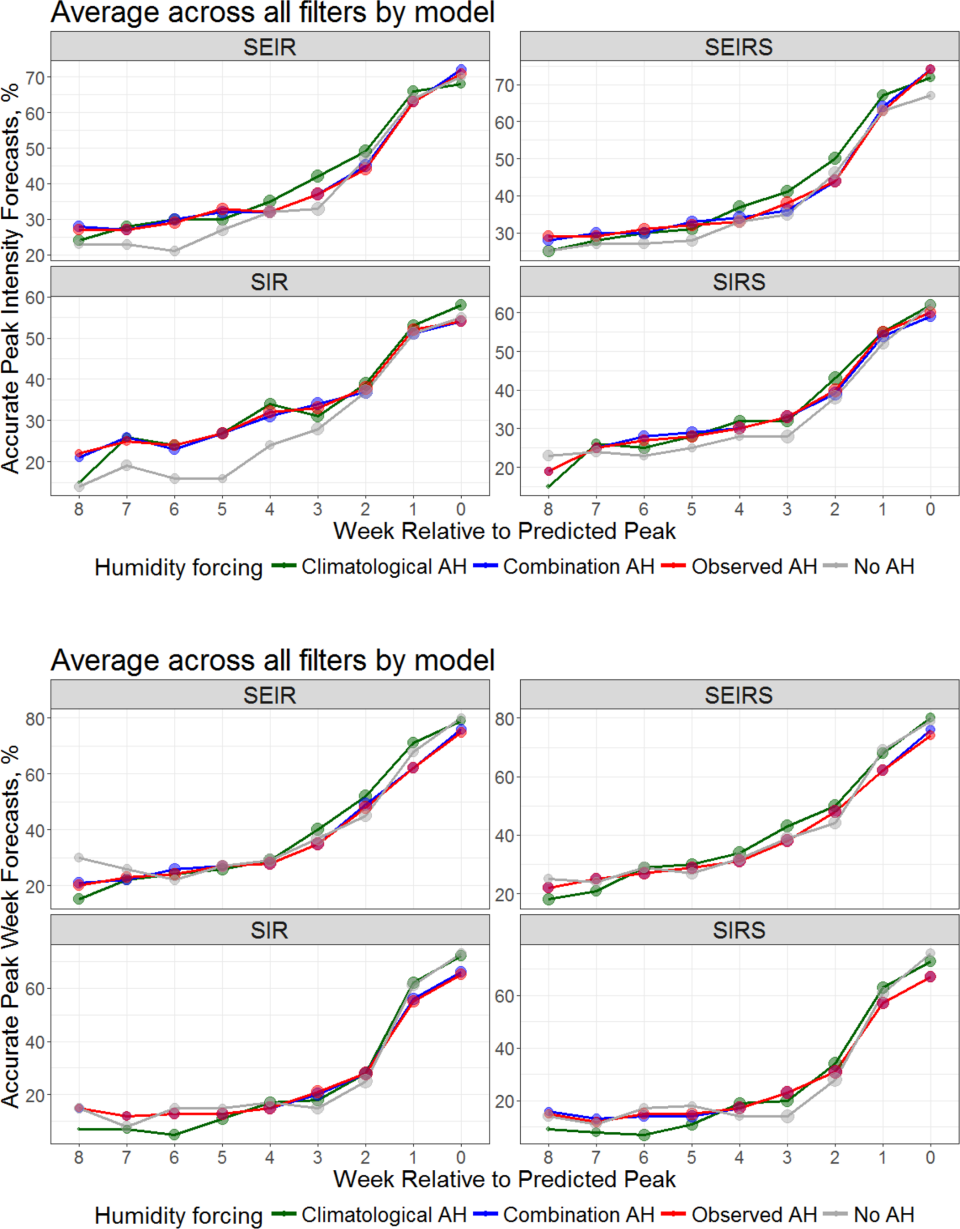
Percentage of forecasts accurate for predictions of peak intensity (top, within ±25% of observed peak intensity) and peak timing (bottom, within ±1 week of the observed peak) plotted as a function of forecast lead relative to the predicted peak for each of the 4 models forms (SEIR, SEIRS, SIR and SIRS). Shown are the forecast accuracies for models with climatological AH forcing (green), observed AH forcing (red), a combination of observed AH during optimization and climatological AH during forecast (blue), and no AH forcing (grey). The number of forecasts (log transformed) at each lead is represented by the size of the dot.

When we stratify the forecasts by model form, we find similar results for peak intensity (**Fig 4**). Specifically, the no AH forecasts are less accurate than the 3 AH-forced forecasts for each of the 4 compartmental model forms. Similarly, the forecasts of peak week stratified by model form are similar to the overall findings (**Fig 1**).

**Fig 5:**
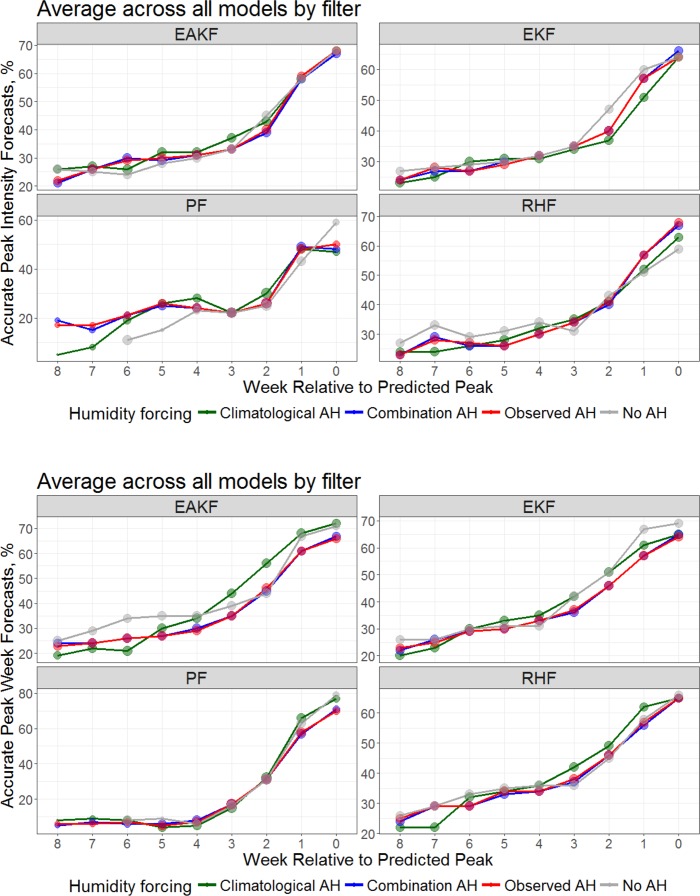
Percentage of forecasts accurate for predictions of peak intensity (top, within ±25% of observed peak intensity) and peak timing (bottom, within ±1 week of the observed peak) plotted as a function of forecast lead relative to the predicted peak for each of the 4 filters (EAKF, EnKF, PF and RHF). Shown are the forecast accuracies for models with climatological AH forcing (green), observed AH forcing (red), a combination of observed AH during optimization and climatological AH during forecast (blue), and no AH forcing (grey). The number of forecasts (log transformed) at each lead is represented by the size of the dot.

For peak intensity, stratification by filter reveals a less clear distinction between the no AH forecasts and the 3 AH-forced forecasts (**Fig 5**). The EAKF and EnKF no AH peak intensity forecasts do comparatively well for 0- to 2-week leads, whereas the RHF no AH forecasts do well for 4- to 8-week leads. The PF no AH forecasts of peak intensity generally perform less well at all leads but 0 weeks. For peak week, the results by filter are less distinct. Here the EAKF no AH forecasts perform better at longer lead times (5–8 weeks), and the EnKF at 0 to 1 week leads.

## Discussion

Overall, our findings indicate that peak intensity forecast accuracy improves with some type of AH forcing regardless of lead time. With the exception of long-lead time forecasts, where the low number of samples for the no AH approach make it difficult to interpret the results, forecast of peak week timing is also generally improved with AH forcing. Analyses of forecast error reveal that predictions generated with no AH forcing are most likely to perform worse than counterpart AH-forced prediction. While this tendency is consistent for forecast peak intensity, for peak timing, RMSE2 and RMSE4, the no AH approach at times produced superior forecasts (Figs 2 and 3), although the overall performance of the no AH form was worse (Table 1).

Among the 3 AH forcing approaches the differences were less clear. The climatological AH approach most often performed best (Table 1); however, for forecast of peak intensity and peak timing, the accuracy of the climatological and combination AH approaches was not statistically different (Table 2). It is interesting that forecasts with climatological AH forcing were more accurate than those generated using observed AH conditions. This finding indicates that the short-term fluctuations of observed AH due to synoptic variability—the 1–4 day variability associated with storms and frontal systems—may actually degrade prediction. Whether this effect is due to the transience of the synoptic AH signal, which corrupts filter optimization, ILI+ observational noise, or the simplicity of the models, which may not appropriately represent the effects of these fluctuations on virus transmissibility, is not clear.

To test whether model simplicity underlies our findings, we performed a synthetic test in which we used local daily, observed AH data for 2003–2015 and the SIRS model to generate time series of influenza incidence for 61 cities (see S1 Text). Daily cases were aggregated by calendar week and observations were drawn from a negative binomial distribution. These synthetic observations were then used, in turn, in conjunction with the EAKF, EnKF and RHF filters to optimize the SIRS model, with each of the 4 AH approaches, and generate forecasts of the synthetic truth for each of the 61 cities and 10 seasons. Forecast results for this synthetic target were similar to the findings for the ILI+ target (S1 and S2 Tables). In particular, forecasts made using the climatological AH approach most consistently ranked first or tied for first even though observed AH had been used to generate the synthetic observations. This finding indicates that model simplicity is not the primary reason for our findings.

For shorter lead predictions (1 to 4 weeks) the improvement of the climatological AH approach over other approaches appears to be greater for the 2 models that include a latent period (i.e. the SEIR and SEIRS models, see Fig 4). Laboratory evidence and physical considerations point to an immediate effect of ambient humidity on virus survival and transmissibility [4]. However, given that there exists an intrinsic delay between infection and seeking medical attention, a lag between humidity anomalies and observed changes in rates of ILI and influenza positivity rates should be evident, and has similarly been observed for excess pneumonia and influenza mortality [18]. For ILI+, observations are summed over a calendar week, so some individuals are no doubt infected and seek clinical care in the same week; however, for other individuals, the effects of humidity at the end of one week, would be expected to modulate ILI+ levels during the following week [43]. It thus may be that the models with a latent period (i.e. SEIR and SEIRS) are able to capture some of this lag—but perhaps for the wrong reason in that the period of latency accounts for some of the time between infection and seeking medical attention.

While there is no perfect estimate of influenza infection rates, the use of 2003–2015 historical GFT municipal ILI estimates in this study may have introduced some biases. Firstly, Google repeatedly altered their algorithm for estimating ILI [44–45]; we here used GFT ILI estimates as they were initially made available in real time, rather than subsequently revised estimates, in order to produce retrospective forecasts as they would have been generated in real time. Secondly, GFT ILI national and regional estimates have documented inaccuracies relative to target CDC ILI measurements [46–47]. Thirdly, GFT ILI estimates were developed and validated using national and regional CDC ILI targets. Detailing of the precise algorithm used by Google or the process of extrapolation to sub-regional spatial scales has not been published. Our use of municipal ILI estimates, which are spatially correlated and derived from the same GFT algorithm, suggests that the 95 cities used in this study may not provide 95 independent tests of the importance of humidity. Consequently, it is possible that the significance of the rank differences reported in Table 2 are inflated.

In general, our findings support the continued use of climatological AH forcing when generating influenza forecasts. This approach has been our primary means for generating operational real-time forecasts. Many other groups are also presently developing and generating influenza forecasts using a combination of dynamical and/or statistical methodologies [48–52], both of which have their strengths. Given the findings here, which indicate that inclusion of humidity forcing improves forecast accuracy, this forcing should be included and tested in conjunction with other forecasting systems. Further, as new, improved model forms and filtering methods are brought online, the effects of humidity forcing on forecast accuracy will need to be continually monitored.

## Supporting information

S1 Data 2003–2015 historical GFT estimates of municipal ILI, as these data were released in real time. (CSV)

S1 Text Description of synthetic testing procedure and findings. (DOCX)

S1 Table Mean Friedman ranks of forecast error for predictions of synthetic truth targets of peak intensity, peak week and incidence during the first 2 weeks (RMSE2) and 4 weeks (RMSE4) of forecast. For pairwise tests of significance see **S2 Table**. Best performing model forms are in bold. Note, two forms may be best if not statistically different.
(DOCX)

S2 Table Pairwise p-values derived from Nemenyi tests of the synthetic forecast ranks shown in S1 Table. Asterisks designate differences significant at p<0.01 (**) and p<0.001 (***).
(DOCX)

S1 Fig Example forecasts from Atlanta, GA during 2005–2006. Shown are the average trajectories across all 16 model-filter combinations for each of the 4 AH forcings. Successive weekly forecasts are shown for Weeks 45–60. Climatological AH forecasts are shown in red; combination AH forecasts in green; observed AH forecasts in blue; and no AH forecasts in purple. ILI+ observations are denoted by the black triangles and the vertical dashed line delimits the forecast initiation week and the observations used to optimize models.
(TIFF)

S2 Fig Example forecasts from New York city during 2007–2008. Shown are the average trajectories across all 16 model-filter combinations for each of the 4 AH forcings. Successive weekly forecasts are shown for Weeks 45–64. Climatological AH forecasts are shown in red; combination AH forecasts in green; observed AH forecasts in blue; and no AH forecasts in purple. ILI+ observations are denoted by the black triangles and the vertical dashed line delimits the forecast initiation week and the observations used to optimize models.
(TIFF)

S3 Fig Example forecasts from Tampa Bay, FL during 2005–2006. Shown are the average trajectories across all 16 model-filter combinations for each of the 4 AH forcings. Successive weekly forecasts are shown for Weeks 45–64. Climatological AH forecasts are shown in red; combination AH forecasts in green; observed AH forecasts in blue; and no AH forecasts in purple. ILI+ observations are denoted by the black triangles and the vertical dashed line delimits the forecast initiation week and the observations used to optimize models.
(TIFF)

S4 Fig Percentage of forecasts accurate for predictions of peak intensity using various error margins to define accuracy. Shown are the forecast accuracies as a function of week relative to predicted peak for models with climatological AH forcing (green), observed AH forcing (red), a combination of observed AH during optimization and climatological AH during forecast (blue), and no AH forcing (grey). The top left sub-panel shows accuracy per the error margins specified in the main manuscript.
(TIFF)

S5 Fig Percentage of forecasts accurate for predictions of peak week using various error margins to define accuracy. Shown are the forecast accuracies as a function of week relative to predicted peak for models with climatological AH forcing (green), observed AH forcing (red), a combination of observed AH during optimization and climatological AH during forecast (blue), and no AH forcing (grey). The top left sub-panel shows accuracy per the error margins specified in the main manuscript.
(TIFF)
