# Supplementary material for: The use of ambient humidity conditions to improve influenza forecast
Source: PLoS Comput Biol. 2017 Nov 16;13(11):e1005844. doi: 10.1371/journal.pcbi.1005844 (PMC5708837; doi:10.1371/journal.pcbi.1005844)
Supplement: S1 Text — (DOCX) [file pcbi.1005844.s002.docx]

S1 Text

The use of ambient humidity conditions to improve influenza forecast

Jeffrey Shaman^1^, Sasikiran Kandula^1^, Wan Yang^1^, Alicia Karspeck^2^

^1^Department of Environmental Health Sciences, Mailman School of Public Health, Columbia University, New York, NY 10032

^2^National Center for Atmospheric Research, Boulder, CO 80305

**Supporting Text**

*Synthetic Tests*

We used model-generated (‘synthetic’) data to further evaluate the relative accuracy of the 4 AH forcing approaches. These synthetic data provide a model-generated ‘truth’ against which forecast accuracy can be evaluated while controlling for model misspecification. That is, our compartmental model forms are simplified representations of actual influenza transmission dynamics; this simplification likely produces forecast error. By using the same core compartmental model both to generate synthetic truths and forecast that truth, we can account for the effects of model misspecification. Further, we were specifically interested in examining whether the finding that forecast with climatological AH forcing generally outperformed forecast with observed AH forcing was robust. By using a synthetic truth generated with observed AH forcing, we can examine whether this finding holds when model misspecification is not an issue and the type of AH forcing has been prescribed.

We therefore used local daily, observed AH data for 2003-2015 and the SIRS model to generate time series of influenza incidence for 61 cities. Daily cases (i.e. the synthetic truth) were aggregated by calendar week and observations were drawn from a negative binomial distribution. These synthetic observations were then used, in turn, in conjunction with the EAKF, EnKF and RHF filters to optimize the SIRS model, with each of the 4 AH forcing approaches, and generate forecasts of the synthetic truth for each of the 61 cities and 10 seasons. Forecast results for this synthetic target were similar to the findings for the ILI+ target (S1-S2 Tables). In particular, forecasts made using the climatological AH approach most consistently ranked first or tied for first even though observed AH had been used to generate the synthetic observations.

Specifically, the observed AH-forced forecasts had the lowest ranked mean error for peak intensity (S1 Table); however, pairwise comparison showed that this ranking was not statistically distinct from the climatological AH-forced forecasts, whereas all other pairs were statistically different (S2 Table). For error in peak week and RMSE during the first 2 and 4 weeks of forecast, climatological AH forcing had the lowest ranked mean error (S1 Table) and was statistically superior to the other 3 AH forcing approaches (S2 Table). These findings indicate that climatological AH-forced forecasts produced statistically identical or more accurate forecasts of synthetic truth targets generated with observed AH forcing. It suggests that the high-frequency variations present in observed daily AH may corrupt filter optimization and that smoother, climatological AH forcing enables more accurate prediction.
