# Supplementary material for: The use of ambient humidity conditions to improve influenza forecast
Source: PLoS Comput Biol. 2017 Nov 16;13(11):e1005844. doi: 10.1371/journal.pcbi.1005844 (PMC5708837; doi:10.1371/journal.pcbi.1005844)
Supplement: S1 Table — For pairwise tests of significance see S2 Table. Best performing model forms are in bold. Note, two forms may be best if not statistically different. (DOCX) [file pcbi.1005844.s003.docx]

**Table S1.** Mean Friedman ranks of forecast error for predictions of synthetic truth targets of peak intensity, peak week and incidence during the first 2 weeks (RMSE2) and 4 weeks (RMSE4) of forecast. For pairwise tests of significance see **Table S2**. Best performing model forms are in bold. Note, two forms may be best if not statistically different.

| **Forecast** | **Peak Intensity** | **Peak Week** | **RMSE2** | **RMSE4** |
| --- | --- | --- | --- | --- |
| **Climatological AH** | **2.28** | **1.87** | **2.11** | **2.16** |
| **Combination** | 2.56 | 2.04 | 2.46 | 2.40 |
| **No AH** | 2.70 | 2.53 | 3.06 | 3.07 |
| **Observed AH** | **2.20** | 1.99 | 2.36 | 2.36 |
