# Supplementary material for: The use of ambient humidity conditions to improve influenza forecast
Source: PLoS Comput Biol. 2017 Nov 16;13(11):e1005844. doi: 10.1371/journal.pcbi.1005844 (PMC5708837; doi:10.1371/journal.pcbi.1005844)
Supplement: S2 Table — Asterisks designate differences significant at p<0.01 (**) and p<0.001 (***). (DOCX) [file pcbi.1005844.s004.docx]

**Table S2.** Pairwise p-values derived from Nemenyi tests of the synthetic forecast ranks shown in Table S1. Asterisks designate differences significant at p<0.01 (**) and p<0.001 (***).

| **Peak Intensity Forecasts** | | | |  | **Peak Week Forecasts** | | | |
| --- | --- | --- | --- | --- | --- | --- | --- | --- |
|  | **Climatological** | **Combination** | **No AH** |  |  | **Climatological** | **Combination** | **No AH** |
| **Combination** | <0.001*** | - | - |  | **Combination** | <0.001*** | - | - |
| **No AH** | <0.001*** | 0.001** | - |  | **No AH** | <0.001*** | <0.001*** | - |
| **Observed** | 0.537 | <0.001*** | <0.001*** |  | **Observed** | <0.001*** | 0.058 | <0.001*** |
|  | | | | | | | | |
| **RMSE during the first 2 weeks of Forecast (RMSE2)** | | | |  | **RMSE during the first 4 weeks of Forecast (RMSE4)** | | | |
|  | **Climatological** | **Combination** | **No AH** |  |  | **Climatological** | **Combination** | **No AH** |
| **Combination** | <0.001*** | - | - |  | **Combination** | <0.001*** | - | - |
| **No AH** | <0.001*** | <0.001*** | - |  | **No AH** | <0.001*** | <0.001*** | - |
| **Observed** | <0.001*** | <0.001*** | <0.001*** |  | **Observed** | <0.001*** | 0.415 | <0.001*** |
